# The Cognitive and Mood-Related Costs of Loneliness: Why Marital Status Matters in Old Age

**DOI:** 10.3390/geriatrics10050117

**Published:** 2025-08-26

**Authors:** Maristella Belfiori, Francesco Salis, Benedetta Puxeddu, Antonella Mandas

**Affiliations:** 1 Department of Medical Sciences and Public Health, University of Cagliari, 09042 Cagliari, Italyamandas@unica.it (A.M.); 2 Department of Biomedical Sciences, University of Cagliari, 09042 Cagliari, Italy; 3 Department of Internal Medicine, University Hospital “Azienda Ospedaliero-Universitaria” of Cagliari, 09042 Cagliari, Italy

**Keywords:** aging, comprehensive geriatric assessment, marital status, cognitive decline, depressive symptom

## Abstract

**Background:** The 21st century is characterized by a significant and ongoing rise in the aging population across Europe. In this context, marital status may act as a relevant social factor influencing health trajectories in later life. This study explores the association between marital status and various health-related outcomes in community-dwelling older adults. **Methods:** We enrolled 1201 patients ≥ 65 years (median age: 81, interquartile range (IQR): 76–84) attending the Geriatric Outpatient Service at the University Hospital of Cagliari. Each participant underwent a Comprehensive Geriatric Assessment (CGA). **Results:** Married individuals were significantly less likely to report depressive symptoms (Risk Ratio (RR) = 0.82; 95% Confidence Interval (CI): 0.73 to 0.92; *p* = 0.0004) and had a 1.26-point reduction in Geriatric Depression Scale (GDS) scores (β = –1.26; 95% CI: −2.03 to −0.50; *p* = 0.0013). Separate/Single participants exhibited significantly higher Mini-Mental State Examination (MMSE) scores (β = 1.60; 95% CI: 0.19 to 3.01; *p* = 0.0262). In contrast, Widowed individuals showed significantly poorer cognitive performance (RR = 1.12; 95% CI: 1.02 to 1.23; *p* = 0.0204), with lower MMSE scores (β = −1.10; 95% CI: −2.08 to 0.12; *p* = 0.0279). They also had a higher likelihood of depressive symptoms (RR = 1.16; 95% CI: 1.04 to 1.30; *p* = 0.0072) and a 1.19-point increase in GDS scores (β = 1.19; 95% CI: 0.38 to 1.99; *p* = 0.0039). **Conclusions:** Although observational design precludes causal inference, our findings highlight the significance of marital status as a social factor associated with cognitive function and mood in older adults. Integrating this dimension into the CGA may enhance its ability to capture social vulnerabilities in later life.

## 1. Introduction

Over the last century, Europe has undergone a significant demographic transformation characterized by a steady increase in the proportion of older adults in the population. In Italy, the Italian National Institute of Statistics (ISTAT) confirms the intensification of this trend with the proportion of elderly citizens (65+) expected to reach 34.6% by 2050 [1,2,3]. 

The aging population presents new challenges for healthcare systems [4]: while the overall health status of older adults has improved over the last century due to better socioeconomic conditions [5], the increasing burden of multimorbidity, polypharmacy, and frailty places substantial pressure on health services [4,5,6]. 

Parallel to the rise in health needs, there is growing concern regarding the availability and adequacy of social support networks for older adults [7,8]. Notably, caregiving is often provided informally by family members, many of whom are themselves elderly and face their limitations. Despite their central role in long-term care, informal caregivers frequently lack adequate training and resources, which can lead to caregiver burden and suboptimal care [9,10]. Moreover, structural gaps in community support services, including limited availability of elder day-care centers, further exacerbate the vulnerabilities of this population. 

Notably, in recent decades, marital patterns among older adults have undergone significant changes. In the United States, marital instability in later life has increased markedly, with divorce rates among individuals over 50 having doubled since the 1990s [10,11]. Similarly, in Italy, recent ISTAT data confirm a persistent decline in marriage rates alongside a parallel rise in marital instability [12]. Globally, widowhood remains a prevalent marital status among older adults. In Italy alone, over 3.6 million individuals aged 65 and older are widowed [12]. According to recent ISTAT data, nearly one-in-five older adults is at risk of social isolation, frequently as a result of spousal bereavement [13,14]. These demographic shifts underscore the need to better understand how marital status may influence aging processes and health outcomes. 

In this epidemiologic context, the present study aims to investigate the association between marital status and cognitive, affective, functional, mobility, and nutritional outcomes in a large cohort of community-dwelling older adults evaluated through a Comprehensive Geriatric Assessment (CGA). These findings may offer valuable insights into the social determinants of health in later life.

## 2. Materials and Methods

### 2.1. Participants 

This cross-sectional study was conducted with a convenience sample of 1201 patients, recruited from the Geriatric Outpatient Service at the University Hospital of Cagliari between January 2020 and December 2024. 

### 2.2. Inclusion/Exclusion Criteria

Patients aged 65 years or older were included in this study, and all of them underwent a CGA. Patients younger than 65 years, those lacking anamnestic data, and individuals who did not provide informed consent were excluded from participation.

### 2.3. Assessment

Trained geriatricians collected information on patients’ marital status (categorized into three groups: Married, Separated/Single, and Widowed), living conditions, and family networks to assess their need for assistance from a family member or caretaker. Additionally, demographic data, including sex, previous employment, and years of education, were gathered. We also recorded details about current or past smoking habits, as well as whether the patient was a current or former alcohol consumer.

Comorbidities were assessed using the Cumulative Illness Rating Scale (CIRS), which includes 14 medical conditions: arterial hypertension; vascular, respiratory, gastrointestinal, hepatic, renal, and genitourinary diseases; ophthalmological and otolaryngologic disorders; musculoskeletal, dermatological, neurological (excluding dementia), endocrine–metabolic, and psychiatric–behavioral conditions. The enrolled patients underwent a CGA, which included cognitive, mood, functional, mobility, and nutritional assessments. Cognitive function was assessed employing two primary screening instruments:

- The Mini-Mental State Examination (MMSE) [15], which evaluates orientation, memory, attention, language, and visual construction: a score of 23 or lower typically indicates cognitive impairment [16]. We adjusted the MMSE scores based on education levels and age to avoid false positives.
- The Clock Drawing Test (CDT) [17], which assesses verbal understanding, memory, and spatial knowledge: a cutoff score of 6 identifies cognitive deficits.

We used the 15-item Geriatric Depression Scale (GDS) for mood assessment [18], which requires “yes” or “no” responses. Scores range from 0 (no depressive symptoms) to 15 (significant mood deflection). A score over 5 suggests the presence of depressive symptoms.

The assessment of the functional and mobility domains was conducted using the following tools:

- The Activities of Daily Living (ADLs) scale [19,20], which evaluates a person’s ability to perform essential daily activities like personal hygiene, dressing, toileting, mobility (walking, sitting, standing, lying down, climbing stairs), continence, and eating: the scale ranges from 0 to 100, where 0 indicates complete dependence and 100 is full autonomy.
- The Instrumental Activities of Daily Living (IADLs) scale [19,20], which measures an individual’s ability to perform complex daily activities that support home and community life, such as using the telephone, managing finances, shopping, and preparing food: scores range from 0 (complete dependence) to 8 (complete autonomy).
- The Performance-Based Physical Test (PPT) [21], which provides an objective assessment of physical function in the elderly through simulated daily activities: scores above 20 indicate no disability, 2–19 moderate disability, and 10 or less severe disability.
- The Tinetti Performance-Oriented Mobility Assessment (POMA) [22], a tool for evaluating balance and gait in older adults, scoring from 0 to 28: a score below 19 indicates a high fall risk, while a score above 24 implies a low fall risk.

We utilized the Exton-Smith Scale (ESS) [23], a 5-item questionnaire for assessing the risk of pressure sore development. It evaluates key factors such as physical and mental health, activity levels, mobility, and incontinence occurrences. Each item is scored from 1 to 4, with a total score below 13 indicating a higher risk of pressure lesions. 

For nutritional assessment, we used the Mini-Nutritional Assessment (MNA) [24], which includes (1) anthropometric measurements like body mass index, midarm and calf circumferences, and weight loss; (2) lifestyle factors and mobility; (3) meal frequency, dietary intake, and feeding autonomy; and (4) individual health perceptions. A score below 17 suggests an inadequate nutritional status.

### 2.4. Statistical Analysis

The Shapiro–Wilk test was employed to assess the variables’ distribution. Given the evidence of an asymmetric distribution, quantitative variables were expressed as median and interquartile range (IQR) or as percentages, where appropriate. Quantitative variables were compared using the Mann–Whitney U test, while categorical variables were analyzed using the Chi-square test. These tests were applied in univariate analyses to explore differences in assistance (from a caretaker or a family member), alcohol consumption, smoking status, demographic parameters (age, education), and clinical test scores (MMSE, CDT, GDS, ADLs, IADLs, PPT, POMA, ESS, MNA) across marital status categories (Married, Separated/Single, Widowed) and by sex within each marital status group.

A Directed Acyclic Graph (DAG) was constructed to identify potential confounders for multivariable analysis. Multivariable modeling was performed using Log-Binomial Regression. When Log-Binomial Regression did not converge, Poisson regression with Huber–White robust variance correction was applied. Additionally, we performed Quantile Regression, an extension of linear regression, not adopted due to the non-normality of residuals, as determined by the D’Agostino–Pearson test. 

The following variables, as dependent ones, were analyzed in both multivariable models and used with the specified dichotomization in the Log-Binomial Regression:

- MMSE: “Absence of cognitive impairment” for scores ≥24, and “Cognitive impairment” for scores <24;
- CDT: “Absence of cognitive impairment” for scores ≥7, and “Cognitive impairment” for scores <7;
- GDS: “Absence of mood deflection” for scores ≤5, and “Mood deflection” for scores >5;
- ADLs: “Absence of moderate/severe dependence” for scores ≥91, and “Moderate/severe dependence” for scores <91;
- IADLs: “Absence of dependence” for scores ≥6, and “Dependence” for scores <6;
- PPT: “Absence of disability” for scores ≥21, and “Presence of disability” for scores <21;
- POMA: “Absence of fall risk” for scores ≥25, and “Presence of fall risk” for scores <25;
- ESS: “Absence of risk for pressure ulcers” for scores ≥13, and “Risk for pressure ulcers” for scores <13;
- MNA: “Satisfactory nutritional status” for scores ≥24, and “Risk of malnutrition” for scores <24.

In both multivariable models, the three variables representing marital status (Married, Separated/Single, and Widowed) were used as independent variables, with each marital status dichotomized as follows: the absence of each marital status was coded as 0, and its presence as 1. Furthermore, the sex variable was categorized as independent, with a binary classification in which 1 denoted Women and 0 denoted Men. According to the DAG model, the identified confounders included sex, education, and age. Log-Binomial Regression results were reported as Risk Ratios (RRs) with 95% Confidence Intervals (CIs), while Quantile Regression findings were presented as effect sizes (β) with corresponding *p*-values (*p*). A significance threshold of *p* ≤ 0.05 was applied, with results interpreted in the context of the 95% CI. We utilized Forest Plots to illustrate the results of Log-Binomial Regressions and a Heatmap to visualize the findings of Quantile Regressions. Statistical analyses were performed using RStudio software (version 4.4.2). Additional details, including the DAG model, descriptive distributions by sex, and extended multivariate analyses, are provided in the Supplementary Materials (Figures S1 and S2 and Tables S1 and S2).

## 3. Results

### 3.1. Overview of Patients’ Characteristics

The study involved 1201 patients aged 65 and older (median age: 81, IQR: 76–84), of whom 818 (68.1%) were women and 82 (6.8%) were aged 90 years or older. The characteristics of the study population are summarized in Table 1. 

Out of 1201 patients, 253 (21.1%) had an education level below 3 years. Additionally, 728 patients (60.6%) exhibited pathological MMSE scores, while 818 patients (68.1%) had pathological CDT scores. Among the cohort, 200 patients (16.65%) displayed severe mood deflection on the GDS. 

A total of 425 patients (35.2%) were identified as having previously engaged in homemaking activities, while 1024 patients (85.3%) presented with musculoskeletal and dermatological disorders (Figure S2). Moreover, nearly 95% of the patients, amounting to 1148 individuals, reported having more than three distinct disorders.

### 3.2. Sex-Based Differences

Regarding sex differences (Table 1 and Figure S2), women were predominantly Widowed (53.8%) and more frequently identified as homemakers (50.9%). They also more often received assistance from family members (χ^2^ = 11.17, *p* = 0.0006), had lower educational attainment (*p* < 0.0001), scored higher on the GDS (*p* < 0.0001), and showed greater dependence in ADLs (*p* = 0.0003). Conversely, men were more likely to be Married (72.1%) or employed as clerks (9.4%) or farmers (9.4%) and reported more frequently as current (χ^2^ = 11.82, *p* = 0.0006) or former smokers (χ^2^ = 210.631, *p* = 0.386). Men also exhibited greater dependence in IADLs (*p* < 0.0001). No other statistically significant differences were observed between sexes.

### 3.3. Sex Differences Within Marital Status Groups and Marital Status Group Comparisons: Univariate Analysis 

In line with the study’s aims, we stratified the entire sample based on marital status, identifying 572 Married (47.6%), 126 Separated/Single (10.5%), and 503 Widowed individuals (41.9%). Univariate analyses were performed to compare: (i) women vs. men within each marital status group and (ii) each marital status group vs. all other participants combined (e.g., Widowed vs. Not-Widowed). Table 2 reports only the statistically significant results for these comparisons.

### 3.4. Marital Status-Based Differences: Multivariate Analysis

#### 3.4.1. Log-Binomial Regression

Multivariate Log-Binomial Regressions were conducted to evaluate the association between marital status and key outcomes, adjusting for age, sex, and education based on a DAG-informed model. For MMSE, only sex was included as a covariate due to prior score correction for age and education.

In terms of cognitive performance (MMSE), being Widowed was associated with a higher likelihood of impairment (RR = 1.12; 95% CI: 1.02 to 1.23; *p* = 0.020). No significant associations were observed for the Married or Separated/Single groups, although the latter showed a trend toward better performance (RR = 0.86; 95% CI: 0.72 to 1.00, *p* = 0.083). For depressive symptoms (GDS), Married individuals showed a significantly lower risk (RR = 0.82; 95% CI: 0.73 to 0.92; *p* = 0.004). Women (RR = 1.22; 95% CI: 1.07 to 1.40, *p* = 0.004) and lower education (RR = 0.97; 95% CI: 0.96 to 0.99, *p* < 0.0010) were also associated with increased risk, while age was not statistically significant. In a separate model comparing Widowed vs. Not-Widowed individuals, widowhood conferred a 16% increased risk likelihood of depressive symptoms (RR = 1.16; 95% CI: 1.04 to 1.30; *p* = 0.007), with similar associations for sex and education.

**Figure 1:**
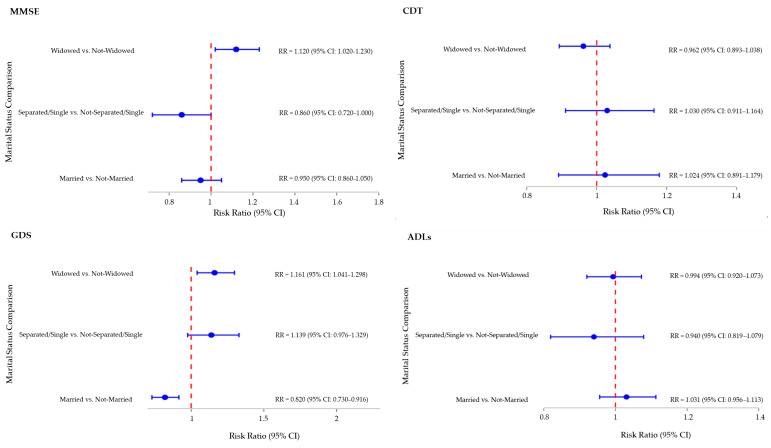

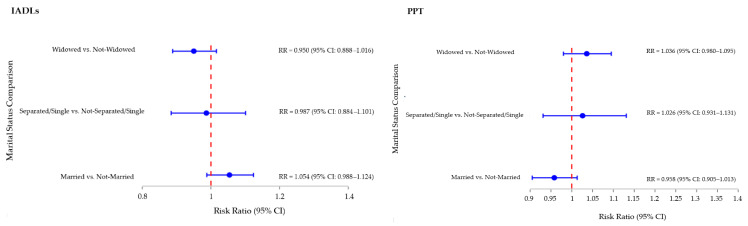

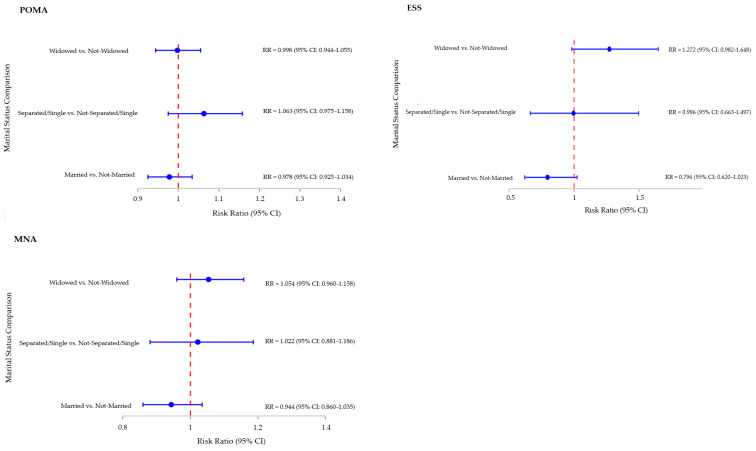
Multivariate Analysis—Log-Binomial Regressions Forest Plot: Association between marital status and performance tests. **Notes:** This figure presents RRs and 95% CIs from Log-Binomial Regression models evaluating the association between marital status and performance on MMSE, CDT, GDS, ADLs, IADLs, PPT, POMA, ESS, and MNA. The Forest Plots represent the Risk Ratio for each marital status category compared with the combination of the other two categories (e.g., Widowed vs. Not-Widowed, where “Not-Widowed” includes both Married and Single/Separated participants). Models were adjusted for age, sex, and education (except for MMSE models, which were adjusted only for sex), which were not included in the Forest Plot. RR values are displayed with 95% Confidence Intervals. Abbreviations: **ADLs**: Activities of Daily Living; **CDT**: Clock Drawing Test; **CI:** Confidence Interval; **ESS**: Exton-Smith Scale; **GDS**: Geriatric Depression Scale; **IADLs:** Instrumental Activities of Daily Living; **MMSE**: Mini-Mental State Examination; **MNA**: Mini-Nutritional Assessment; **POMA:** Tinetti Performance-Oriented Mobility Assessment; **PPT**: Performance-Based Physical Test; **RR:** Risk Ratio.

As shown in Figure 1, no significant associations were found between marital status and performance on other tests, including CDT, ADLs, IADLs, PPT, POMA, ESS, or MNA.

We also conducted comparisons on performance tests using Married patients as the reference group. As reported in Table S1, Separated/Single individuals (RR = 1.24; 95% CI: 1.05 to 1.47; *p* = 0.011) and Widowed individuals (RR = 1.22; 95% CI: 1.07 to 1.41; *p* = 0.001) exhibited a higher likelihood of depressive symptoms than their Married counterparts.

#### 3.4.2. Quantile Regression

**Figure 2:**
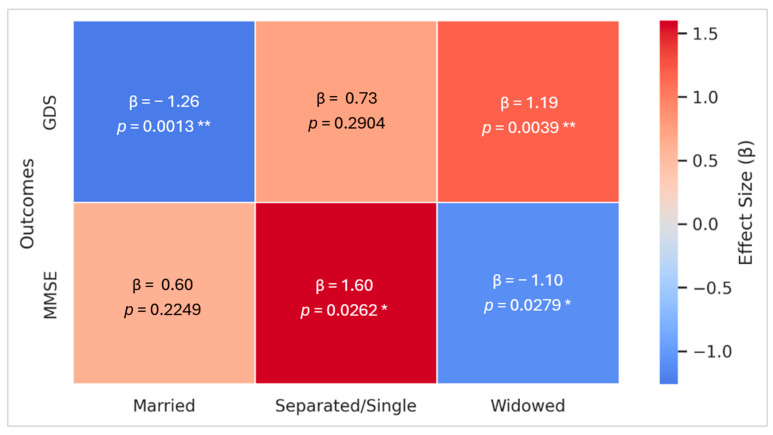
Multivariate Analysis—Quantile Regressions Heatmap: Association between marital status and MMSE and GDS scores. **Notes:** This Heatmap displays effect sizes (β) from multivariate Quantile Regression analyses evaluating the association between marital status and MMSE and GDS scores, adjusted for age, sex, and education (except for MMSE models, which were adjusted only for sex). The Heatmap represents the effect size for each marital status category compared with the combination of the other two categories (e.g., Widowed vs. Not-Widowed, where “Not-Widowed” includes both Married and Single/Separated participants). Confounders and other outcome measures (CDT, ADLs, IADLs, PPT, POMA, EES, and MNA) are not represented in this figure. β values are displayed within each cell. Asterisks denote statistical significance levels: * *p* < 0.05, ** *p* < 0.01. Abbreviations: **β**: effect size; **GDS**: Geriatric Depression Scale; **MMSE**: Mini-Mental State Examination.

Additionally, a Quantile Regression analysis adjusted for potential confounders was performed (Figure 2). Concerning cognitive performance (MMSE), Separated/Single individuals showed significantly higher median MMSE scores (β = 1.60; 95% CI: 0.19 to 3.01; *p* = 0.026), whereas Widowed patients exhibited significantly lower scores (β = −1.10; 95% CI: −2.08 to 0.12; *p* = 0.028). No significant effects were observed for marital status or sex.

Regarding mood (GDS), being Married was associated with significantly milder depressive symptoms (β = −1.26, 95% CI: −2.03 to −0.50; *p* = 0.001), whereas widowhood correlated with a significant increase (β = 1.19, 95% CI: 0.38 to 1.99; *p* = 0.004). In subgroup analyses, among Married participants, female (β = 1.50, 95% CI: 0.72 to 2.27; *p* = 0.0001) and lower education (β = −0.18, 95% CI: −0.28 to −0.08; *p* = 0.0004) exhibited higher GDS scores, while age showed a non-significant trend (β = −0.06,95% CI: −0.11 to 0.00; *p* = 0.07). Among Widowed individuals, female (β = 1.56, 95% CI: 0.79 to 2.33; *p* = 0.0007), lower education (β = −0.17, 95% CI: −0.26 to −0.08; *p* = 0.0004), and age (β = −0.06,95% CI: −0.12 to −0.00; *p* = 0.039) were all significantly associated with increased depressive symptoms. No significant associations were found between marital status and other outcomes, including CDT, ADLs, IADLs, PPT, POMA, ESS, and MNA.

We also compared Married patients, using them as a reference group, with Separated and Widowed patients on performance tests. Widowhood tended to be associated with a poorer MMSE performance (β = −1.00, 95% CI: −2.04 to 0.04 *p* = 0.060) and was significantly associated with higher depressive symptom scores (β = 1.34, 95% CI: 0.50 to 2.18; *p* = 0.002) compared with marriage. Detailed results are presented in Table S2.

## 4. Discussion

Our study provides a comprehensive evaluation of community-dwelling older adults, suggesting that, beyond clinical variables, social context, particularly marital status, might shape various health-related outcomes. Our cohort reflects broader demographic trends in Europe, with a high median age (81 years) and a substantial proportion of participants aged over 90. According to the literature [25,26,27], longer life expectancy is often accompanied by a high burden of chronic diseases; indeed, nearly 95% of our participants had at least three comorbidities. As the global population ages, the demand for caregiving is rising, creating challenges for healthcare systems. In our study, the vast majority (72.8%) required caregiving support, primarily provided by family members (46.6%), especially among women (77.5%) and the Widowed (52.9%), reaffirming the central role of informal caregiving in community-based elder care [8,9].

Women represented nearly 70% of the sample, consistent with the so-called “sex-frailty paradox,” in which women live longer despite greater health impairments [28,29,30]. Although women generally accumulate more health deficits than men throughout their lifespan, the rate of deficit accumulation is slower. Consequently, women exhibit a higher prevalence of multiple health deficits at advanced ages, primarily attributable to their longer life expectancy [29,30]. The observed imbalance also reflects gendered health-seeking behaviors, with older women being more likely to engage in outpatient care, while older men may underutilize services due to stigma or traditional norms [31,32].

Our findings suggest that marital status may influence certain health behaviors. For example, Married individuals, especially men, were more likely to be former smokers, possibly reflecting the protective influence of a partner in promoting health behavior change [33]. Conversely, Married men showed greater alcohol use, diverging from the existing literature that links marriage to reduced alcohol intake [34]. These discrepancies may be influenced by cultural norms that normalize alcohol consumption in Married men. 

A key finding concerns the cognitive domain and mood. Widowed individuals showed significantly poorer cognitive performance (+12% likelihood of a pathological MMSE score; −1.10-point MMSE median score), aligning with previous studies reporting increased Alzheimer’s risk and higher β-amyloid levels in Widowed individuals [35,36,37,38,39]. In contrast, Separated/Single individuals presented better cognitive performance, with a 1.6-point increase in median scores, though the literature remains inconclusive [40,41,42]. This finding may reflect greater cognitive flexibility or openness to new experiences among these individuals, potentially influencing both relationship status and cognitive performance. Furthermore, the challenges of separation may foster problem-solving skills, while increased engagement in social or cognitively stimulating activities and factors associated with cognitive preservation could also play a protective role in cognition [43]. However, the heterogeneity of the group, which includes both never married and divorced individuals, requires cautious interpretation, as their different social contexts may have distinct impacts on cognitive outcomes.

Marital status was also associated with mood. Widowed individuals showed a 16% increased likelihood of depressive symptoms and a 1.19-point higher GDS score, whereas Married participants exhibited a 19% lower likelihood and a 1.26-point reduction in depressive symptoms. These findings are in line with prior research supporting marriage’s potential role in mental health, possibly due to greater emotional and social support [44,45,46], and the heightened risk of depression among Widowed or unmarried older adults [47].

Interestingly, despite the previous literature suggesting a beneficial effect of marriage on cognitive function [48], neither Log-Binomial nor Quantile Regression found a significant association between being Married and MMSE or CDT performance. This may reflect unmeasured confounders such as relationship quality, social participation, or limitations in the sensitivity of the cognitive screening tools used. Moreover, marital status showed no association with functional outcomes, mobility measures, risk of pressure ulcers, or nutritional status, possibly related to a selection bias that likely underrepresents frailer, bedridden, or institutionalized individuals typically associated with a worse physical and nutritional status [49,50]. The exclusion of these more vulnerable subgroups may have limited the variability and severity of impairments in functional and nutritional domains, thereby reducing the statistical power to detect any potential associations with marital status. Additionally, a subgroup analysis of Separated/Single individuals, due to their heterogeneity, is required to confirm and better understand the associations observed in this combined category.

From a person-centered care perspective, these findings offer several practical implications:

- Integration of social determinants such as marital status into the CGA: Understanding an individual’s relational environment can help develop more-tailored care strategies, particularly for mood and cognitive well-being.
- Addressing social isolation and relational loss: Public health initiatives and community programs (e.g., elder day-care centers, peer-support networks) may help mitigate the psychosocial effects of spousal loss or absence of close relationships in later life.
- Policy relevance: Findings support the need for policies that recognize caregiving as a structural component of geriatric health systems. This may include financial incentives, caregiver training programs, and integration of social services into medical pathways.

This study is strengthened by its multidimensional geriatric assessment in a large sample of community-dwelling older adults. However, its cross-sectional nature precludes causal inference. The predominantly female and outpatient-based sample may also limit the generalizability of findings to the broader older adult population. Additionally, we lacked measures of relationship quality beyond marital status, and this limitation prevents us from capturing the potentially crucial role of marital quality in the observed associations. Future research incorporating qualitative and longitudinal data is needed to better understand how relational contexts influence aging-related outcomes. 

## 5. Conclusions

Our findings suggest that marital status may be correlated with key aspects of cognitive function and mood in older adults. Specifically, widowhood was associated with worse cognitive performance and higher depressive symptoms, whereas Separated/Single individuals were associated with better MMSE scores and being Married with lower mood deflection. Although causal relationships cannot be established due to the observational design, these associations underscore the importance of considering marital status as a relevant social determinant in the context of CGA. Future healthcare strategies should integrate such social dimensions to better the support systems of older adults, consistent with the principles of person-centered care.

## Figures and Tables

**Table 1:** Characteristics of the study sample.

*Variable*	*Total Sample* *(1201)*	*Male* *(383, 31.9%)*	*Female* *(818, 68.1%)*	*Married* *(572, 47.6%)*	*Separated/Single* *(126, 10.5%)*	*Widowed* *(503, 41.9%)*
***Marital Status,*** ***n (%)***						
*Married*	572 (47.6%)	276 (72.1%)	293 (35.8%)			
						
*Separated/Single*	126 (10.5%)	43 (11.2%)	85 (10.4%)			
						
*Widowed*	503 (41.9%)	64 (16.7%)	440 (53.8%)			
***Assistance,*** ***n (%)***						
*From a* *caretaker*	314 (26.1%)	89 (23.2%)	225 (27.5%)	143 (25%)	37 (29.4%)	134 (26.6%)
						
*From a family member*	560 (46.6%)	151 (39.4%)	409 (50%)	245 (42.8%)	49 (38.9%)	266 (52.9%)
***Alcohol Consumption,*** ***n (%)***						
*Current consumers*	55 (4.6%)	40 (10.4%)	15 (1.8%)	34 (5.9%)	6 (4.8%)	15 (3%)
						
*Former consumers*	41 (3.4%)	30 (7.8%)	11 (1.3%)	26 (4.5%)	8 (6.3%)	7 (1.4%)
***Smoking Status,*** ***n (%)***						
*Current smokers*	79 (6.6%)	39 (10.2%)	40 (4.9%)	45 (7.9%)	8 (6.3%)	26 (5.2%)
						
*Former smokers*	335 (27.9%)	212 (55.4%)	123 (15.0%)	206 (36%)	42 (33.3%)	87 (17.3%)
***Demographic Parameters*** ***and*** ***Test Scores***						
*Age (years)*	81 (76–85)	81 (76–85)	81 (77–85)	80 (75–83)	78 (73–83)	83 (79–87)
						
*Education (years)*	5 (4–8)	7 (5–8)	5 (4–8)	5 (5–8)	6 (5–8)	5 (3–8)
						
*MMSE*	22.3 (16.7–25.7)	22.7 (17.8–25.8)	22.0 (16.5–25.7)	22.5 (16.6–26)	23.4 (18.4–26.2)	21.5 (16.5–25.4)
						
*CDT*	4 (3–7)	5 (3–7)	3 (3–6)	4 (3–7)	5 (3–7)	3 (2–5.5)
						
*GDS*	6 (4–10)	5 (2–8)	7 (4–10)	5 (3–9)	7 (4–10)	8 (4–10)
						
*ADLs*	81 (65–93)	85 (65–96)	79 (65–92)	83 (66–95)	87 (71–97)	77 (62–90)
						
*IADLs*	3 (1–5)	2 (1–5)	3 (1–5)	3 (1–5)	4 (1–6)	3 (1–5)
						
*PPT*	13 (9–18)	14 (9–20)	12 (8–18)	14 (9–20)	14 (10–20)	11 (8–17)
						
*POMA*	17 (11–22.25)	18 (12–24)	17 (11–22)	18 (12–24)	18 (12–23)	16 (10–21)
						
*ESS*	15 (13–18)	16 (14–18)	15 (13–17)	16 (13–18)	16 (14–18)	15 (12–17)
						
*MNA*	22.5 (19.5–24.5)	22.5 (19.5–25.0)	22.0 (19.5–24.5)	22.5 (20–25)	22.5 (18.75–25)	22 (19.5–24)

**Notes**: Data are presented as n (%) or median (IQR). Abbreviations: **ADLs**: Activities of Daily Living; **CDT**: Clock Drawing Test; **ESS**: Exton-Smith Scale; **GDS**: Geriatric Depression Scale; **IADLs:** Instrumental Activities of Daily Living; **MMSE**: Mini-Mental State Examination; **MNA**: Mini-Nutritional Assessment; **POMA:** Tinetti Performance-Oriented Mobility Assessment; **PPT**: Performance-Based Physical Test.

**Table 2:** Univariate analysis: Sex differences within marital status groups and marital status group comparisons.

Variable	Widowed (M vs. F)	Separated/Single (M vs. F)	Married (M vs. F)	Widowed (W) vs. Not-Widowed (NW)
***Former Smoker*** ***n (%)***	M: 35 (54.7%) F: 52 (11.8%) χ^2^ = 71.53 *p* < 0.0001	—	M: 159 (57.6%) F: 47 (15.9%) χ^2^ = 107.74 *p* < 0.0001	—
***Current Alcohol Use *** ***n (%)***	M: 7 (10.9%) F: 19 (1.8%) χ^2^ = 16.01 *p* = 0.0001	—	M: 29 (10.5%) F: 5 (1.7%) χ^2^ = 19.83 *p* < 0.0001	—
***Former Alcohol Use *** ***n (%)***	M: 3 (4.7%) F: 4 (0.9%) χ^2^ = 5.79 *p* = 0.016	—	M: 21 (7.6%) F: 5 (1.7%) χ^2^ = 11.51 *p* = 0.001	—
				
***Care By Family*** ***n (%)***	—	—	M: 100 (36.2%) F: 145 (49.0%) χ^2^ = 9.47 *p* = 0.002	W: 266 (52.9%) NW: 294 (42.1%) χ^2^ = 13.60 *p* = 0.002
***Education*** ***(Median, IQR)***	M: 8 (5–9) F: 5 (3–6) *p* < 0.0001	—	M: 6.5 (5–8) F: 5 (4.5–8) *p* < 0.0001	—
***MMSE*** ***(Median, IQR)***	—	M: 21.35 (15.3–25.4) F: 24.4 (19.62–26.47) *p* = 0.010	—	W: 21.5 (16.5–25.4) NW: 22.7 (17.4–26.0) *p* = 0.010
***CDT*** ***(Median, IQR)***	—	—	M: 5 (3–7) F: 3 (3–6) *p* = 0.008	W: 3 (2–5.5) NW: 5 (3–5) *p* = 0.003
***GDS*** ***(Median, IQR)***	M: 6 (2.75–6) F: 8 (4–11) *p* = 0.007	—	M: 5 (2–7) F: 6.5 (4–10) *p* < 0.0001	W: 8 (4–10) NW: 6 (3–9) *p* < 0.0001
				
***ADLs*** ***(Median, IQR)***	M: 84.5 (61.5–94.0) F: 77 (62–90) *p* = 0.049	—	—	W: 77 (67–96) NW: 83 (62–90) *p* < 0.0001
***IADLs*** ***(Median, IQR)***	—	—	M: 2 (1–5) F: 3 (2–6) *p* < 0.0001	—
***PPT*** ***(Median, IQR)***	—	—	—	W: 11 (8–17) NW: 14 (9–20) *p* < 0.0001
***POMA*** ***(Median, IQR)***	—	—	—	W: 16 (10–21) NW: 18 (12–23) *p* = 0.000003

**Notes**: Comparisons between groups were performed using the Mann–Whitney U test for quantitative variables and the Chi-square test for categorical variables. Only statistically significant comparisons are reported in the table. Abbreviations: **ADLs**: Activities of Daily Living; **CDT**: Clock Drawing Test; **ESS**: Exton-Smith Scale; **F:** females; **GDS**: Geriatric Depression Scale; **IADLs:** Instrumental Activities of Daily Living; **IQR:** interquartile range **M:** males; **MMSE**: Mini-Mental State Examination; **MNA**: Mini-Nutritional Assessment; **NW:** Not Widowed; **POMA:** Tinetti Performance-Oriented Mobility Assessment; **PPT**: Performance-Based Physical Test; **W:** Widowed.

## Data Availability

The data presented in this study are available on request from the corresponding author due to privacy reasons.

## Supplementary Materials

The following supporting information can be downloaded at: https://www.mdpi.com/article/10.3390/geriatrics10050117/s1: Figure S1: Directed Acyclic Graph (DAG): The relationships between marital status and multivariate analysis outcomes; Figure S2: Principal distribution of employment status and diseases in total sample and by sex; Table S1: Multivariate analysis—Log-Binomial Regressions: Comparison of test performance between Married (reference group), Separated/Single, and Widowed participants; Table S2: Multivariate Analysis—Regressions: Comparison of test performance between Married (reference group), Separated/Single, and Widowed participants.

## Abbreviations

The following abbreviations are used in this manuscript:

ADLs	Activities of daily living
CDT	Clock drawing test
CGA	Comprehensive geriatric assessment
CI	Confidence interval
CI	Confidence intervals
CIRS	Cumulative illness rating scale
DAG	Directed acyclic graph
β	Effect sizes
ESS	Exton-Smith scale
GDS	Geriatric depression scale
IADLs	Instrumental activities of daily living
IQR	Interquartile range
ISTAT	Italian National Institute of Statistics
MMSE	Mini-mental state examination
MNA	Mini-nutritional assessment
PPT	Performance-based physical test
RR	Risk ratio
RRs	Risk ratios
POMA	Tinetti performance-oriented mobility assessment
