# Supplementary material for: The Cognitive and Mood-Related Costs of Loneliness: Why Marital Status Matters in Old Age
Source: Geriatrics (Basel). 2025 Aug 26;10(5):117. doi: 10.3390/geriatrics10050117 (PMC12452690; doi:10.3390/geriatrics10050117)
Supplement: Supplementary file 1 [file geriatrics-10-00117-s001.zip › geriatrics-3796704-supplementary.pdf]

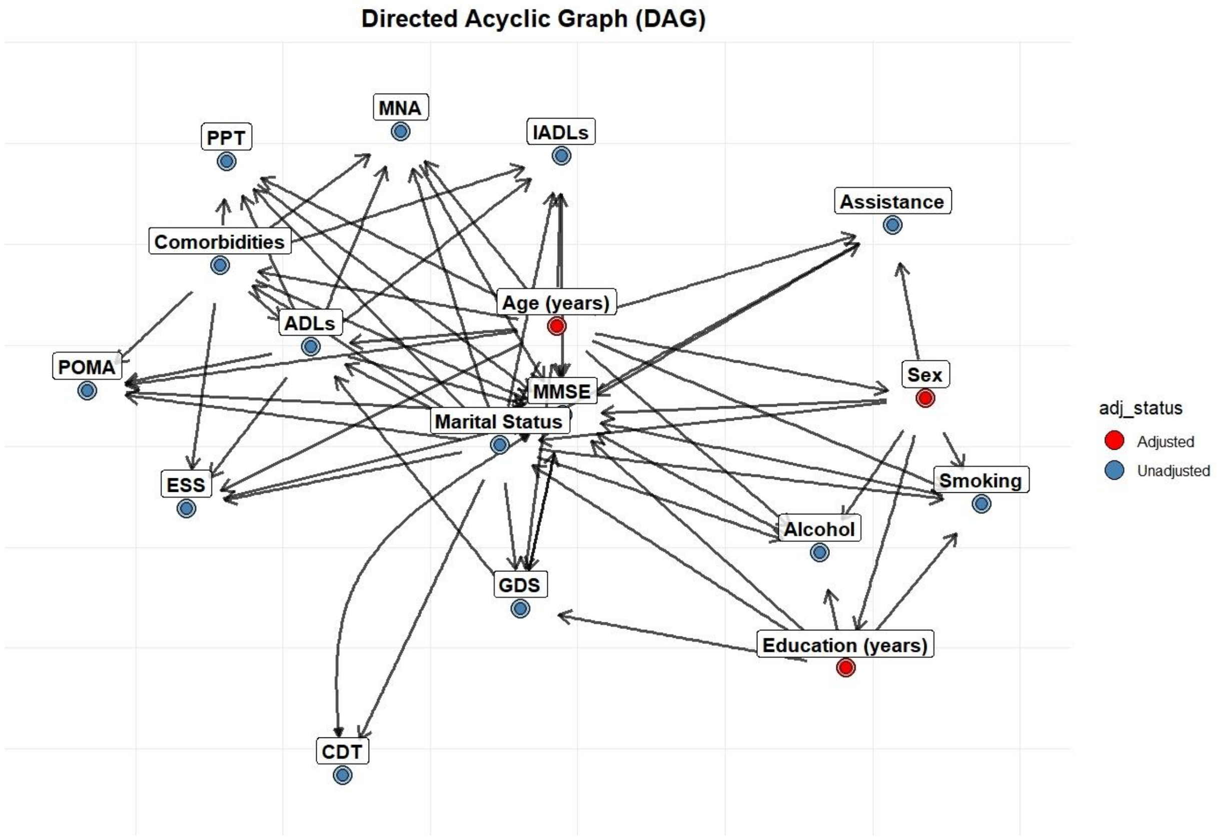

Figure S1. Directed Acyclic Graph (DAG): The relationships between marital status and multivariate analysis outcomes.

Notes: To identify potential confounding variables in the relationship between Marital Status (exposure variable) and Outcomes included in the multivariable analysis (MMSE, CDT, GDS, ADLs, IADLs, PPT, POMA, ESS, MNA), we constructed a Directed Acyclic Graph (DAG) based on theoretical knowledge and existing literature. The DAG visually represents the causal relationships among the variables considered in the study, including Age, Sex, Education, Smoking habits, Alcohol consumption and Comorbidities. Analysis of the DAG indicated that Age, Sex and Education level are the main confounders (indicated in red). The figure illustrates the DAG using the MMSE as outcome. Abbreviations: **ADLs**: Activities of Daily Living; **CDT**: Clock Drawing Test; **DAG**: Directed Acyclic Graph; **ESS**: Exton-Smith Scale; **GDS**: Geriatric Depression Scale; **IADLs:** Instrumental Activities of Daily Living; **MMSE**: Mini-Mental State Examination; **MNA**: Mini Nutritional Assessment; **POMA:** Tinetti Performance-Oriented Mobility Assessment; **PPT**: Performance-Based Physical Test.

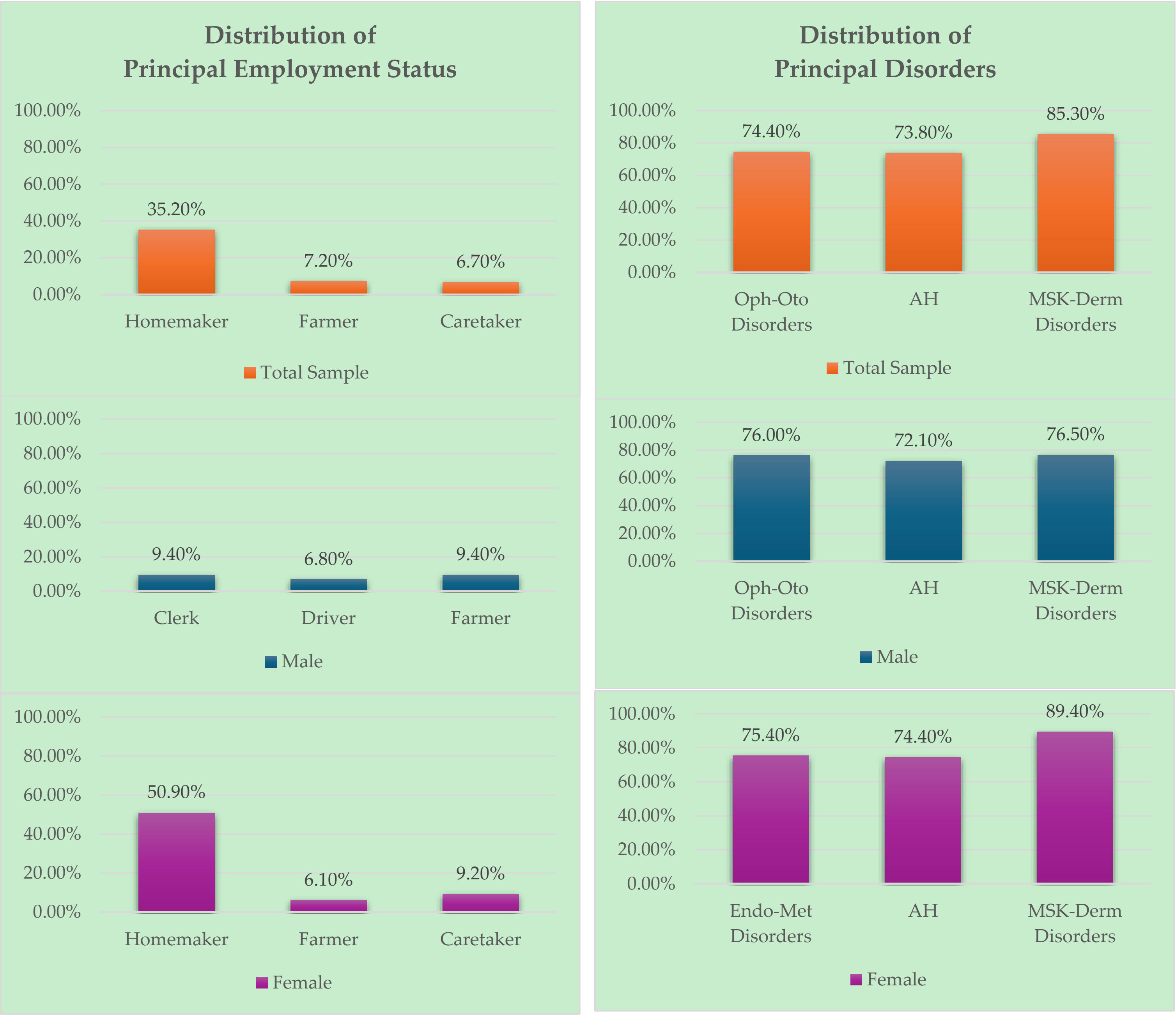

Figure S2. Principal distribution of employment status and diseases in total sample and by sex.

Abbreviations: **AH**: Arterial Hypertension; **MSK-Derm Disorders**: Musculoskeletal and Dermatological Disorders; **Oph-Oto Disorders**: Opthalmological and Otolaryngologic Disorders; **Endo-Met Disorders:** Other Endocrine-Metabolic Disorder.

Table S1. Multivariate analysis—Log-Binomial Regressions: Comparison of test performance between Married (reference group), Separated/Single, and Widowed participants.

Outcome	Variables	RR	95% CI	*p*-value
MMSE	*Intercept*	*0.58*	*0.53, 0.64*	*0.138*
	Separated	0.91	0.76, 1.09	0.256
	Widowed	1.10	0.99, 1.21	0.128
	Women	1.05	0.94, 1.17	0.490
	Age (years)	/	/	/
	Education (Years)	/	/	/
CDT	*Intercept*	*0.18*	*0.10, 0.32*	*<0.0001*
	Separated/Single	1.01	0.89, 1.15	0.865
	Widowed	0.96	0.89, 1.04	0.562
	Women	*1.11*	*1.02, 1.21*	*0.121*
	Age (years)	*1.02*	*1.01, 1.02*	*<0.0001*
	Education (Years)	*0.96*	*0.95, 0.97*	*<0.0001*
GDS	Intercept	0.79	0.38, 1.61	0.512
	Separated/Single	*1.24*	*1.05, 1.47*	*0.011*
	Widowed	*1.22*	*1.08, 1.38*	*0.001*
	Women	*1.23*	*1.07, 1.41*	*0.003*
	Age (years)	0.99	0.99, 1.00	0.256
	Education (Years)	*0.97*	*0.96, 0.99*	*0.001*
ADLs	*Intercept*	*0.10*	*0.06, 0.18*	*<0.0001*
	Separated/Single	0.93	0.81, 1.07	0.337
	Widowed	0.98	0.91, 1.06	0.636
	Women	*1.12*	*1.03, 1.23*	*0.007*
	Age (years)	*1.02*	*1.01, 1.03*	*<0.0001*
	Education (Years)	*0.96*	*0.96, 0.98*	*<0.0001*
IADLs	*Intercept*	*0.24*	*0.15, 0.37*	*<0.0001*
	Separated/Single	0.96	0.86, 1.08	0.526
	Widowed	0.94	0.88, 1.01	0.101
	Women	*0.92*	*0.86, 0.99*	*0.017*
	Age (years)	*1.02*	*1.01, 1.02*	*<0.0001*
	Education (Years)	*0.97*	*0.96, 0.98*	*<0.0001*
PPT	*Intercept*	*0.31*	*0.21, 0.46*	*<0.0001*
	Separated/Single	1.04	0.94, 1.16	0.401
	Widowed	1.04	0.98, 1.11	0.146
	Women	1.05	0.98, 1.12	0.158
	Age (years)	*1.01*	*1.01, 1.02*	*<0.0001*
	Education (Years)	*0.98*	*0.96, 0.98*	*<0.0001*
POMA	*Intercept*	*0.39*	*0.26, 0.57*	*<0.0001*
	Separated/Single	1.07	0.97, 1.17	0.163
	Widowed	1.01	0.95, 1.07	0.752
	Women	*1.10*	*1.03, 1.17*	*0.006*
	Age (years)	*1.01*	*1.00, 1.01*	*<0.0001*
	Education (Years)	*0.97*	*0.96, 0.98*	*<0.0001*
ESS	*Intercept*	*0.04*	*0.01, 0.24*	*<0.001*
	Separated/Single	1.12	0.73, 1.71	0.606
	Widowed	1.30	0.99, 1.70	0.058

	Women	1.00	0.76, 1.31	0.987
	Age (years)	*1.02*	*1.01, 1.04*	*0.029*
	Education (Years)	*0.93*	*0.89, 0.96*	*<0.0001*
MNA	*Intercept*	*0.30*	*0.16, 0.57*	*<0.001*
	Separated/Single	1.05	0.90, 1.22	0.551
	Widowed	1.06	0.96, 1.17	0.221
	Women	1.03	0.93, 1.14	0.602
	Age (years)	*1.01*	*1.01, 1.02*	*0.005*
	Education (Years)	*0.98*	*0.96, 0.99*	*<0.001*

Notes: This table presents RRs and 95% CIs from Log-Binomial regression models evaluating the association between performance on MMSE, CDT, GDS, ADLs, IADLs, PPT, POMA, ESS and MNA, and Marital Status, using Married patients as reference group. Italic indicates statistically significant associations, defined according the 95% CIs. Abbreviations: **ADLs**: Activities of Daily Living; **CDT**: Clock Drawing Test; **CI:** Confidence Interval; **ESS**: Exton-Smith Scale; **GDS**: Geriatric Depression Scale; **IADLs:** Instrumental Activities of Daily Living; **MMSE**: Mini-Mental State Examination; **MNA**: Mini Nutritional Assessment; **POMA:** Tinetti Performance-Oriented Mobility Assessment; **PPT**: Performance-Based Physical Test; **RR:** risk ratio.

Table S2. Multivariate Analysis—Regressions: Comparison of test performance between Married (reference group), Separated/Single, and Widowed participants.

**Outcome**	**Variable**	**Effect sizes (β)**	**Std. Error**	**95% CI**	***p*-value**
MMSE	*Intercept*	*22.80*	*0.46*	*21.90, 23.70*	*<0.0001*
	Separated/Single	1.00	0.74	−0.45, 2.45	0.180
	Widowed	*−1.00*	*0.53*	*−2.04, 0.04*	*0.060*
	Women	−0.40	0.55	−1.48, 0.68	0.466
	Age (years)	/	/		/
	Education (Years)	/	/		/
CDT	Intercept	14.05	1.61	10.89, 17.21	<0.0001
	Separated/Single	−0.07	0.28	−0.62, 0.48	0.797
	Widowed	0.42	0.25	−0.07, 0.91	0.095
	Women	*−0.68*	*0.27*	*−1.21, −0.15*	*0.011*
	Age (years)	*−0.13*	*0.02*	*−0.17, −0.09*	*<0.0001*
	Education (Years)	*0.21*	*0.03*	*0.15, 0.27*	*<0.0001*
GDS	*Intercept*	*10.54*	*2.59*	*5.46, 15.62*	*<0.0001*
	Separated/Single	1.16	0.71	−0.23, 2.55	0.104
	Widowed	*1.34*	*0.43*	*0.50, 2.18*	*0.002*
	Women	*1.49*	*0.40*	*0.71, 2.27*	*0.0002*
	Age (years)	−0.06	0.03	−0.12, 0.00	0.072
	Education (Years)	*−0.18*	*0.06*	*−0.30, −0.06*	*0.007*
ADLs	*Intercept*	*139.14*	*9.00*	*121.51, 156.77*	*<0.0001*
	Separated/Single	−0.12	2.51	−5.03, 4.79	0.960
	Widowed	−0.14	1.55	−3.20, 2.92	0.925
	Women	*−4.46*	*1.48*	*−7.36, −1.56*	*0.003*
	Age (years)	*−0.77*	*0.11*	*−0.98, −0.56*	*<0.0001*
	Education (Years)	*1.10*	*0.14*	*0.82, 1.38*	*<0.0001*
IADLs	*Intercept*	*12.17*	*1.36*	*9.50, 14.84*	*<0.0001*
	Separated/Single	0.27	0.32	−0.36, 0.90	0.393
	Widowed	0.21	0.22	−0.23, 0.65	0.333
	Women	*1.00*	*0.22*	*0.57, 1.43*	*0.0001*
	Age (years)	*−0.14*	*0.02*	*−0.18, −0.10*	*<0.0001*

	Education (Years)	*0.19*	*0.03*	*01.13, 0.25*	*<0.0001*
PPT	*Intercept*	*30.83*	*3.37*	*24.22, 37.44*	*<0.001*
	Separated/Single	−0.59	0.69	−1.94, 0.76	0.396
	Widowed	−0.37	0.57	−1.48, 0.74	0.521
	Women	−0.98	0.60	−2.15, 0.19	0.101
	Age (years)	*−0.26*	*0.04*	*−0.34, −0.18*	*<0.0001*
	Education (Years)	*0.62*	*0.07*	*0.48, 0.76*	*<0.0001*
POMA	*Intercept*	*37.27*	*4.10*	*29.22, 45.32*	*<0.0001*
	Separated/Single	−1.15	0.90	−2.91, 0.61	0.201
	Widowed	−0.66	0.67	−1.97, 0.65	0.329
	Women	−1.13	0.71	−2.52, 0.26	0.114
	Age (years)	*−0.27*	*0.05*	*−0.37, −0.17*	*<0.0001*
	Education (Years)	*0.44*	*0.07*	*0.30, 0.58*	*<0.0001*
ESS	*Intercept*	*24.18*	*1.61*	*21.02, 27.34*	*<0.0001*
	Separated/Single	0.15	0.33	−0.50, 0.80	0.651
	Widowed	0.24	0.26	−0.27, 0.75	0.345
	Women	*−0.88*	*0.26*	*−1.39, −0.37*	*0.0009*
	Age (years)	*−0.12*	*0.02*	*−0.16, −0.08*	*<0.0001*
	Education (Years)	*0.21*	*0.03*	*0.15, 0.27*	*<0.0001*
MNA	Intercept	29.11	2.10	*24.99, 33.23*	*<0.0001*
	Separated/Single	0.01	0.42	−0.81, 0.83	0.971
	Widowed	−0.35	0.34	−1.02, 0.32	0.309
	Women	−0.01	0.35	−0.70, 0.68	0.966
	Age (years)	*0.09*	*0.02*	*0.05, 0.13*	*0.0003*
	Education (Years)	*0.12*	*0.04*	*0.04, 0.20*	*0.0044*

Notes: This table presents effect sizes (β), std errors, 95% CIs and p-values from quantile regression analyses evaluating the association between Outcomes (MMSE, CDT, GDS, ADLs, IADLs, PPT, POMA, ESS and MNA, adjusted for age, sex, and education, and Marital Status, using Married patients as the reference group. Italic denotes statistical significance at $p < 0.05$. Abbreviations:**β**: effect size; **ADLs**: Activities of Daily Living; **CDT**: Clock Drawing Test; **CI:** Confidence Interval; **ESS**: Exton-Smith Scale; **GDS**: Geriatric Depression Scale; **IADLs:** Instrumental Activities of Daily Living; **MMSE**: Mini-Mental State Examination; **MNA**: Mini Nutritional Assessment; **POMA:** Tinetti Performance-Oriented Mobility Assessment; **PPT**: Performance-Based Physical Test; **RR:** risk ratio; **Std. Error**: Standard Error
